# ﻿Nomenclatural history of *Megalonyx* Jefferson, 1799 (Mammalia, Xenarthra, Pilosa, Megalonychidae)

**DOI:** 10.3897/zookeys.1195.117999

**Published:** 2024-03-18

**Authors:** Loren E. Babcock

**Affiliations:** 1 School of Earth Sciences, Orton Geological Museum, The Ohio State University, Columbus, OH, 43210, USA The Ohio State University Columbus United States of America

**Keywords:** Ground sloth, Pleistocene, Quaternary, Thomas Jefferson, Virginia, West Virginia

## Abstract

Both authorship and spelling of the extinct giant sloth genus *Megalonyx* and its type species, *M.jeffersonii* (Mammalia, Xenarthra, Pilosa, Megalonychidae), have been inconsistent. The genus-group name has been cited with two different authorships and three dates, and it has been spelled with two different suffixes. The species-group name has been cited with four different authors and dates, and it has been spelled with two different endings. *Megalonyx* Jefferson, 1799 is the first valid use of the genus-group name; the correct original spelling has the –*onyx* suffix. The type species of *Megalonyx* is *Megatheriumjeffersonii* Desmarest, 1822; the correct original spelling has an –*ii* ending. A vernacular word, megalonyx, refers to species classified in the genus *Megalonyx* Jefferson, 1799.

## ﻿Introduction

*Megalonyx* Jefferson, 1799 (Mammalia, Xenarthra, Pilosa, Megalonychidae) was the first genus-group name erected for an extinct vertebrate animal from the United States. This giant ground sloth, which was widespread across North America and now known from more than 180 localities (Semken et al. 2022), is emblematic of the large-mammal fauna of the Pleistocene Epoch of the Quaternary Period. *Megalonyx* ranks among the best known and most widely recognized extinct mammals; it appears in hundreds of scientific, historical, and popular publications, and appears in film and electronic media.

The first publications on *Megalonyx* (Jefferson 1799; Wistar 1799) were among the earliest contributions devoted to the scientific discipline that by the early 1820s would come to be known as “paleontology” (e.g., Simpson 1942; Boyd 1958; Bedini 1985; Rudwick 1997; Rowland 2009; Thomson 2011a, 2011b; De Iuliis 2018). The papers were published long before formal rules of zoological nomenclature were enacted, and standards for nomenclatural acts at the time were weak. These matters have led to misinterpretation or inconsistency about the authorship and correct spelling of *Megalonyx*, and about the authorship and correct spelling of its type species, *M.jeffersonii*. Inconsistency or dispute about these important nomenclatural matters have been a part of the scientific literature, and less formal media sources, from 1799 to the present.

The purpose of this paper is to review the nomenclatural history and authorship of *Megalonyx* and its type species by a review of pertinent early literature. This will serve to eliminate future inconsistency and confusion about the nomenclature of this animal.

## ﻿Discovery and early descriptions

Jefferson (1799), Cuvier (1804a), Leidy (1855), Simpson (1942), Bedini (1985), Stein (1993), Rudwick (1997), Rowland (2009), and Thomson (2011a, 2011b), among others, provided accounts of the discovery of the earliest-known skeletal remains of *Megalonyx*. The bones were collected in 1796 by saltpeter workers from unconsolidated Quaternary sediment in a cave in what was then Greenbriar County, Virginia, USA. The cave was said to belong to Frederic Cromer (Jefferson 1799: 246). Bones were removed from the cave and dispersed among various people as curiosities. From this assortment of skeletal remains, Colonel John Stuart of Virginia sent three bones to Thomas Jefferson, of Monticello, Virginia, in May 1796 (Rowland 2009: 236). Additional bones were supplied to Jefferson later, including by a Mr Hopkins of New York. Jefferson penned a paper on the remains, which in total comprised bones of the left manus, a radius and ulna, and the distal condyles of a femur (Boyd 1958; Rowland 2009; Thomson 2011a). The paper was delivered orally to the American Philosophical Society on 10 March 1797 and published, after emendation, in 1799.

**Figure 1.**
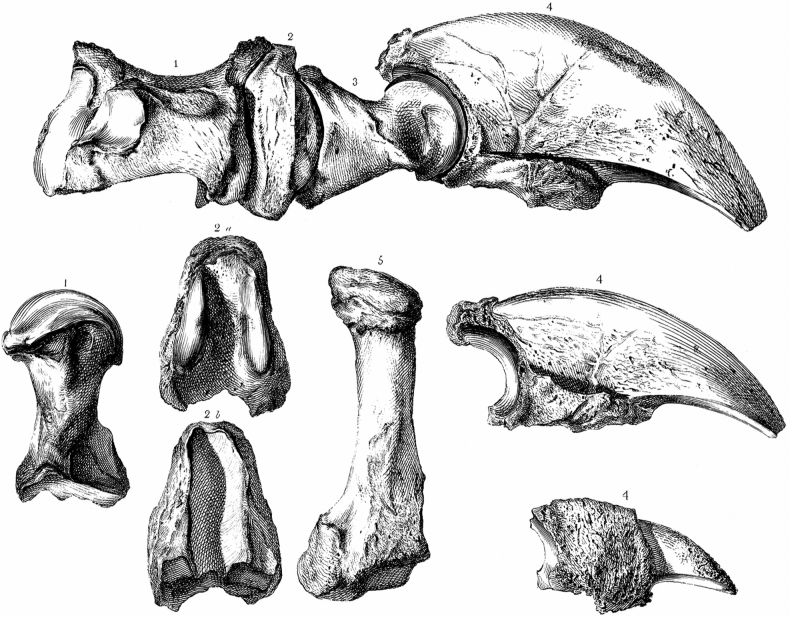
*Megalonyxjeffersonii* (Desmarest, 1822), bones of the holotype, left manus (see Daeschler in Thomson 2011a), reproduced from Wistar (1799: pl. 2, with modification), deposited in the Academy of Natural Sciences of Drexel University, Philadelphia, Pennsylvania (ANSP 12507); Quaternary (Pleistocene), probably from Haynes Cave, Monroe County, West Virginia (*fide*Grady 1997), USA. Wistar’s numbers refer to: 1, 5, metacarpals; 2, 3, phalanges; 4, unguals (claw cores). In the articulated digit at top of figure, the second phalanx (middle bone in the figure) is illustrated upside-down. For scale: the longest ungual, upper right, juxtaposed with other bones of the digit, is 17 cm long.

Jefferson’s (1799) paper describing the skeletal remains included the erection of a genus-group name, *Megalonyx*, to receive them. Accompanying this paper, in the same volume, was a much more detailed analysis and interpretation of the remains, including illustrations, by Caspar Wistar (1799; Fig. 1), who recognized the resemblance of the remains to those of extant sloths. In or before 1804, Georges Cuvier received plaster casts, prepared by Charles Willson Peale, of the described bones, plus some additional remains putatively from the same cave as those reported by Jefferson (1799) and Wistar (1799). Those casts and fossils formed the nucleus for an extensive redescription and interpretation of *Megalonyx* (Cuvier 1804a, reprinted 1812a). Cuvier (1804a, 1812a) confirmed the conclusion of Jefferson (1799) and Wistar (1799) that *Megalonyx* was a sloth based on his comparison with *Megatherium* (Cuvier 1804b, 1812b; see also Bru 1804, 1812).

Some details about the occurrence of remains documented by Jefferson (1799), Wistar (1799), and Cuvier (1804a) were corrected by later authors. Grady (1997) provided cogent evidence that the *Megalonyx* remains described by Jefferson (1799) and Wistar (1799) originated from what is now called Haynes Cave, and which, after political reorganization, is currently located in Monroe County, West Virginia. The owner of the cave in 1796, identified as “Frederick Cromer,” is an apparent misspelling of Frederick Gromer (Grady 1997). Leidy (1855) reported that the tooth (molar) that Cuvier (1804a: fig. 14) illustrated was collected from White Cave, Tennessee, not Frederick Gromer’s cave, as originally reported.

**Figure 2.**
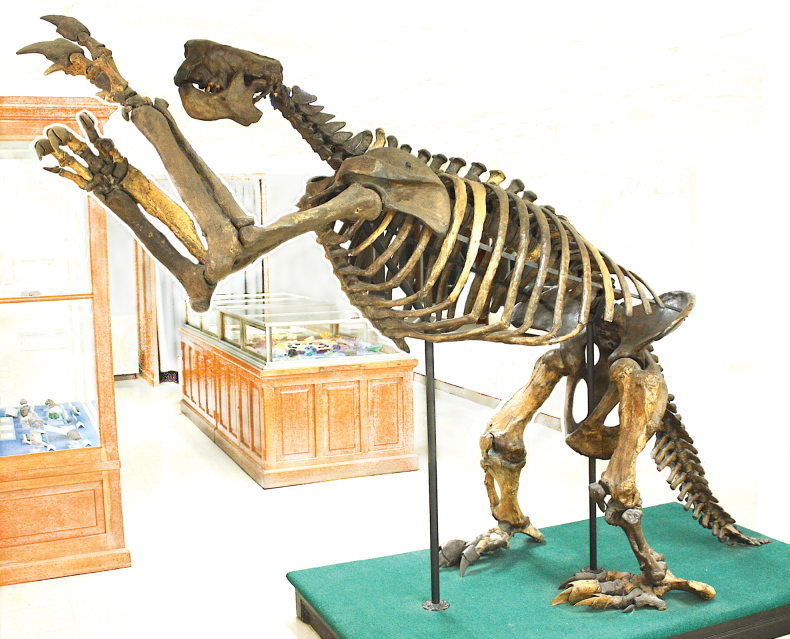
*Megalonyxjeffersonii* (Desmarest, 1822), reconstructed skeleton described by Orton (1891a, 1891b), Claypole (1891), and McDonald et al. (2015), from unconsolidated Quaternary sediment, Millersburg, Ohio, USA; mounted in 1896 by Ward’s Natural Science Establishment for public display in the Orton Geological Museum of The Ohio State University (OSU 15758; see Babcock et al. 2023). The skull is a cast of a specimen illustrated by Leidy (1855: pls I–III, V), with three teeth inserted from the Millersburg megalonyx. As mounted, the skeleton stands 2.1 m tall.

Other early papers that addressed the nomenclature or anatomy of *Megalonyx* include Desmarest (1822), Harlan (1825), Leidy (1855), Orton (1891a), Claypole (1891), and Safford (1892). Many of the early papers citing *Megalonyx* were reviewed by Spamer et al. (1995: 213–216, 308). Desmarest (1822) erected a new species, *Megatheriumjeffersonii* Desmarest, 1822 based on the remains described by Jefferson (1799) and Wistar (1799). Harlan (1825) recombined this species as *Megalonyxjeffersonii* (Desmarest, 1822). Leidy (1855) published the first rather comprehensive description of the *Megalonyx* skeleton, incorporating new morphological information from post-1796 discoveries. Orton (1891a, 1891b) and Claypole (1891) described a partial skeleton from Millersburg, Ohio, that was mounted in 1896 (see McDonald et al. 2015; Babcock et al. 2023), providing an interpretation of the complete skeletal morphology and dimensions of the animal (Fig. 2). Safford (1892) provided a description of the pelvis of *M.jeffersonii* (see also Hovey 1891).

## ﻿Genus-group authorship and spelling

As first published, *Megalonyx* Jefferson, 1799 meets all the requirements for the availability of a new name published after 1757 and before 1931 (International Code of Zoological Nomenclature, Articles 8, 10–12, 21, 50; International Commission on Zoological Nomenclature 2000). This nomenclatural act has priority over all subsequent uses of *Megalonyx* in print (Article 23 of the Code; International Commission on Zoological Nomenclature 2000). The original description includes these components:

1. The nomenclatural act was published using ink on paper (Jefferson 1799: 248).
2. The name was stated to be new (Jefferson 1799: 248).
3. The scientific name, *Megalonyx*, with an uppercase “ *M*,” was accompanied by an etymology, “Great-Claw” (Jefferson 1799: 248). The Linnaean name derives from Greek roots, *megalo*–, great or large, and –*onyx*, claw or fingernail.
4. The scientific name was accompanied by a brief diagnosis; the genus being identified by the “distinguished size of that member,” in reference to the “Great-Claw” (Jefferson 1799: 248).
5. The diagnosis was accompanied by a list and description of skeletal elements (Jefferson 1799: 247–251), plus a table of measurements of the available skeletal material (Jefferson 1799: 248–249).
6. The description was accompanied by a differential comparison with an extant mammal, the African lion (Jefferson 1799: 248–251). Jefferson’s (1799) comparison reflected initial misinterpretation about the affinity of *Megalonyx*, an interpretation that was corrected in a postscript to the paper (Jefferson 1799: 259–260) with reference to *Megatherium* Cuvier, 1796.
7. The geographic and stratigraphic occurrence of the described skeletal elements was identified (Jefferson 1799: 246–247).

Jefferson (1799: 248, 250) introduced two names for the skeletal remains that he described, “Megalonyx” (p. 248), a properly formed, but not italicized, Linnaean name; and “megalonyx” (p. 250) a vernacular equivalent of *Megalonyx*. Ambiguity about the affinity of the animal’s remains (Jefferson 1799: 259–260) is reflected in use of the vernacular term megalonyx, which, with a lowercase “m” and as originally used, is different in form from the Linnaean name. Desmarest (1822: 366) recognized this distinction, indicating its equivalent in French, “Mégathère de Jefferson” (translated as “Jefferson’s megatherium”), as a vernacular name, and separately proposing *Megatheriumjeffersonii* as a Linnaean name. Such a distinction was common in early paleontological publications. Both Wistar (1799: 531) and Cuvier (1804a, 1812a), for example, used “megatherium” as a vernacular term for the ground sloth genus *Megatherium*Cuvier (1796). One of the best-known examples of similar Linnaean and vernacular names is *Mastodon* Cuvier, 1817 as a genus-group name, and mastodon as a vernacular name, for the extinct proboscidean mammal now recognized as *Mammut* Blumenbach, 1799. Similarly, Buckland & Conybeare (in Buckland 1824: 391) applied both a Linnaean genus-group name, *Megalosaurus*, and a vernacular name, megalosaurus, for the first validly named animal that decades later would be called a dinosaur.

Authors (e.g., Boyd 1958; Rudwick 1997, 2005; Rowland 2009; herein, Fig. 2) commonly have used “megalonyx,” or a variant, with a lowercase “m” as a vernacular term for the genus *Megalonyx*. Cuvier (1804a) used the spelling “mégalonix,” in French, but in other articles, which are also in French, he (Cuvier 1804b, 1812a, 1812b) used the spelling “megalonix.” Desmarest (1822), writing in French, spelled the vernacular name as “mégalonyx.” Both Wistar (1799) and Rudwick (1985) used the spelling “megalonix,” in English, for the vernacular term.

Italicizing Latin names was not standard before 1931 (e.g., see Hovey 1891), and in some publication formats, non-italicized names have continued to be used even more recently (as in “Megalonyx”; e.g., Cohen 1995: 63). That Jefferson intended *Megalonyx* to be used as a Linnaean name, however, is evinced in a letter of his, which was reproduced by Faujas-Saint-Fond (1804: 316). In the letter, the name begins with a capital “*M*” and is italicized. However, it is spelled with an –*onix* ending (see discussion below).

*Megalonyx* Jefferson, 1799 was named without designation of a type species or any included species at the time of first publication. As indicated below, Harlan (1825) is deemed to have subsequently designated *Megatheriumjeffersonii* Desmarest, 1822 as the type species of *Megalonyx*. Specifying a type species, or even specifying any species included in a genus, was not always done prior to 1961. One salient example of this involves the dinosaur *Megalosaurus* Buckland & Conybeare in Buckland, 1824. Buckland and Conybeare (in Buckland 1824: 391) announced the genus as new, without naming or including any species in it. The type species, *Megalosaurusbucklandii* Mantell, 1827 was named and designated subsequently by a different author (Mantell 1827: 67–71; see Benson et al. 2008; Howlett et al. 2017).

In a paper that accompanied Jefferson (1799) in the same volume, Wistar (1799) first illustrated, and described in detail, the skeletal materials that Jefferson named as *Megalonyx* (Fig. 1). Wistar’s (1799) analysis lent support for interpretation of *Megalonyx* as a sloth, a view more firmly advocated by Cuvier (1804a, 1812a).

As published, *Megalonyx* Jefferson, 1799, with an –*onyx* suffix is the correct original spelling of the genus-group name (see Article 32 of the Code; International Commission on Zoological Nomenclature 2000). This ending is correctly formed from the Greek root –*onyx*. Some early publications cited this genus with an –*onix* suffix (as *Megalonix*; e.g., Wistar 1799; Faujas-Saint-Fond 1804; Cuvier 1804a, 1804b, 1812a, 1812b). According to Article 33.3 of the Code (International Commission on Zoological Nomenclature 2000), this is an incorrect subsequent spelling.

*Megalonyx* was recognized as a valid genus-group name, with authorship and date stemming from Jefferson (1799) by all 19^th^ and 20^th^ century authors (e.g., Wistar 1799; Faujas-Saint-Fond 1804; Cuvier 1804a, 1812a; Desmarest 1822; Harlan 1825, 1831; Leidy 1855; Cope 1871, 1889; Orton 1891a, 1891b; Claypole 1891; Stock 1925; Osborn 1929, 1935; but see Lyon 1938, discussed below) until the work of Simpson (1942: 162, table), who argued that authorship of *Megalonyx* is “disputed,” that its attribution to Jefferson “is certainly erroneous,” and that Harlan (1825) “may have been the first to use the name in a valid Linnaean form …” Harlan (1825: 201–203), in a compilation of extant and extinct mammals from North America known to the time, however, attributed the Linnaean name *Megalonix* (corrected to *Megalonyx* in an erratum) to Jefferson rather than stating that the name was being newly introduced. Some authors have implicitly accepted Simpson’s (1942) argument and consequently cited Harlan (1825) for authorship of *Megalonyx* (e.g., Hirschfeld and Webb 1968; Hoganson and McDonald 2007; Holte 2012; Semken et al. 2022), whereas others have not (e.g., McDonald 1977; Ward and Allmon 2019). Regardless, Jefferson’s (1799) original use of *Megalonyx* meets the requirements for an available nomenclatural act in all respects (Articles 8, 10–12, 21, 50 of the Code; International Commission on Zoological Nomenclature 2000), which invalidates Simpson’s (1942) suggestion of an alternative authorship and date.

Lyon (1938: 15) attributed the genus *Megalonyx* to Jefferson, but cited 1797, the year the paper was read orally before the American Philosophical Society, as the date of publication. According to Articles 8 and 21 of the Code (International Commission on Zoological Nomenclature 2000), the name became available upon publication in the Society’s Transactions in 1799, not 1797, when the paper was read.

## ﻿Species-group authorship and spelling

Desmarest (1822: 366) first published a species-group name for the skeletal remains that Jefferson (1799) described as *Megalonyx*. Desmarest (1822) placed *Megalonyx* in synonymy with *Megatherium* Cuvier, 1796 and erected the species *Megatheriumjeffersonii* Desmarest, 1822. The skeletal elements of the individual reported by Jefferson (1799) and Wistar (1799) comprise the holotype, by monotypy. The described fossils, which were originally deposited by Jefferson with the American Philosophical Society in 1797, were transferred to the Academy of Natural Sciences of Philadelphia in 1849 (Stein 1993), and they are now deposited in the Academy of Natural Sciences of Drexel University, Philadelphia, Pennsylvania, USA (ANSP 12507, bones of the left manus including metacarpals two, three, and five; the ungual of the first digit; the medial phalanx of the second digit; the proximal and ungual phalanges of the third digit, and the medial and ungual phalanges of the fourth digit, Fig. 1; ANSP 12508, left radius and ulna; see Spamer et al. 1995: 398). Some of the remains illustrated later by Cuvier (1804a, 1812a) also may have derived from this same individual animal, but this has not been unambiguously demonstrated (see discussion of a tooth, Leidy 1855: 4). Multiple *Megalonyx* individuals are known to occur at a few localities (Mercer 1897; Fields 2010; Holte 2011; Semken et al. 2022), including a cave site that possibly served as a maternity den (Holte 2012), so it is conceivable that Haynes Cave has yielded parts of multiple sloth skeletons. In the 19^th^ century, plaster casts of two unguals (claw cores) from the holotype were made widely available for sale by Ward’s Natural Science Establishment, Rochester, New York (Ward 1870, 1891).

Harlan (1825: 26) resurrected the use of *Megalonyx* as a valid genus, which was monospecific at the time of his paper, and recombined the species as *Megalonyxjeffersonii* (Desmarest, 1822). As a result, *Megatheriumjeffersonii* Desmarest, 1822 is the type species of *Megalonyx*, by subsequent designation (Harlan 1825) and monotypy.

With few exceptions, most authors have cited Desmarest (1822) as the first use of the binomen *Megatheriumjeffersonii*. However, Leidy (1855) incorrectly cited Harlan (without a date, but presumably Harlan 1825; see Leidy 1855: 57) as the author of the species, recombined as *Megalonyxjeffersonii*, and this erroneous attribution was followed by Marsh (1874), Orton (1891a, 1891b), and Claypole (1891). Cope (1889: 660) mistakenly attributed authorship of *Megalonyxjeffersonii* to Cuvier (without a date, but presumably Cuvier 1804a). Hirschfeld and Webb (1968: 216) incorrectly cited Wistar (without a date, but undoubtedly Wistar 1799) as the author of *Megalonyxjeffersonii*.

The original spelling of the species-group name, *Megalonyxjeffersonii* (Desmarest, 1822) is with an –*ii* ending. However, some authors have spelled the species name with one –*i* as the ending (as *M.jeffersoni*; e.g., Orton 1891b; Slosson 1927; Lillegraven 1966; Hirschfeld and Webb 1968; Bedini 1985; Saarinen and Karme 2017). According to Articles 32, 33.3, and 33.4 of the Code (International Commission on Zoological Nomenclature 2000), *M.jeffersonii* is the correct original spelling, and substitution of –*i* for –*ii* constitutes an incorrect subsequent spelling.

## ﻿Additional information

### Author ORCIDs

Loren E. Babcock https://orcid.org/0000-0002-9324-9176

### Data availability

All of the data that support the findings of this study are available in the main text.
